# The Genesee Valley Veterinary Medical Association

**Published:** 1898-01

**Authors:** A. George Tegg

**Affiliations:** Secretary


### THE GENESEE VALLEY VETERINARY MEDICAL ASSOCIATION.

THE final preliminary meeting of this Association, to complete organization, was held in Rochester, December 9, 1897, when a suitable constitution and by-laws were presented and accepted, and a committee appointed to secure the proper incorporation of the society. The President delivered an instructive and interesting address. Several of the members exhibited specially interesting specimens. The future meetings will be held semi-annually in January and July. The large number who have identified themselves with the organization, and who are enthusiastic workers, insures the success of the organization, and full reports of the future proceedings will be sent the JOURNAL. A. GEORGE TEGG,
Secretary.

